# Supplementary material for: Development and Reliability of the Oxford Meat Frequency Questionnaire
Source: Nutrients. 2021 Mar 12;13(3):922. doi: 10.3390/nu13030922 (PMC7999625; doi:10.3390/nu13030922)
Supplement: Supplementary file 1 [file nutrients-13-00922-s001.zip › Supplementary File S1.docx]

**Supplementary File S1:** Development process of the Oxford Meat Frequency Questionnaire (MFQ)

#### i) Extracting FSA Food Items and Portion Sizes

To create a comprehensive list of all meat and seafood products consumed in the UK, we extracted all relevant items from the FSA food portion sizes book [1]. Items that we deemed to be umbrella products, such as “white fish, unspecified” were excluded if more specific items were listed (e.g. “cod”). The items were extracted together with their average or medium portion sizes in grams. Where an ‘average’ or ‘medium’ portion size was not provided for an item, we calculated amean average of the ‘small’ and large’ portion size indications, if available. To more accurately represent consumption estimates, we extracted portion sizes exclusive of bone or shell wherever possible. Portion indicators that were likely to be larger than a portion intended for consumption (e.g. the weight of a whole pheasant) were not included. Food items without relevant portion sizes were extracted without a portion size.

#### ii) Creating MFQ Categories

All meat items were groups according to animal source (e.g. beef, chicken and turkey, pork, fish and seafood), commonly consumed product types (e.g. mixed dish, cuts, slices, pies and pastries) and portion size in grams. For instance, the chicken cordon bleu was added to the ‘chicken and turkey animal’ category and grouped with other ‘coated products’ of a similar mean portion weight. Items describing the same product were grouped and the mean of their portion sizes were calculated. In cases where portion sizes were presented in units typically consumed in multiples (e.g. fish fingers) we calculated a number of units which yielded a serving size similar to the rest of the category (e.g. three fish fingers yielded a comparable serving size to one fish burger).

For each animal group, we created a section for ‘mixed dishes’ which encompassed composite mixed dishes containing both meat and non-meat ingredients (e.g. curries, casseroles or stews). To better represent commonly consumed meat products and to improve the accuracy of our portion size estimations, we included separate categories for pies and pastries, sausages, burgers, and offal and liver in all animal groups with exception of fish and seafood. Where there were no FSA items for a specific category (e.g. lamb sausages), we calculated a mean portion size based on data for this category from other animal groups (e.g. beef sausages, pork sausages).

#### iii) Disaggregation of Meat Ingredients

Many of the items contained both meat and non-meat ingredients. To calculate the quantity of meat in each item, we extracted information on the meat content of composite dishes from the food composition database of the NDNS, Year 10 [2]. This database provides information (in g/100g serving) about the meat content of 5923 composite products, detailing the proportion of specific types of meat. We extracted meat content information for all NDNS meat/seafood products which closely reflected items extracted from the FSA food portion size book. The NDNS database covers products using different cooking methods and ingredient compositions, meaning that several NDNS products could match to an FSA item. Where several NDNS products seemed relevant to an FSA item, we extracted all of them. In instances where several NDNS products detailed the same cooking method and the same proportion of meat for one FSA item (e.g. “fish fingers fried in lard” and “fish fingers fried in pufa oil”), only one was included in our final database to avoid duplication and biasing of the overall meat content value. Products containing more than one type of meat (e.g. chicken and prawn paella) were assigned to the animal group with the highest proportion of meat, with quantities of all meat types summed together. For instance, the chicken and prawn paella which contained more chicken than prawn was added to the ‘chicken and turkey’ category, with the quantity of chicken and prawn totalled together. Where there was no meat content data on items for a whole MFQ category, we removed this category from the questionnaire, considering that items not captured by the NDNS dataset are not likely to be commonly consumed.

###### Mixed Dishes Categories

For the mixed dishes section within each animal group, we sorted the relevant NDNS products by meat content and split them into low, medium, and high meat proportions aiming to have near to equal representation in all groups. With this, mixed dishes containing <25% meat were classed as ‘low meat’, dishes containing 25%-40% meat were classed as ‘moderate meat’, and dishes containing >40% meat were classed as ‘high meat’. These three groups were treated as separate MFQ categories.

#### iv) Calculating MFQ Meat Portion Sizes

We calculated mean portion sizes for each MFQ category to provide a single serving size. The mean meat content was first calculated for each food item, and then across all items in a category. Where an FSA item did not have any corresponding NDNS products, the item was still included in the average serving size calculation but skipped in the meat content calculation. To calculate the grams of meat per serving for each MFQ category, the mean serving size for the category was multiplied with its respective mean meat content.

#### v) Creating Serving Size Indicators

Mean serving sizes for each MFQ category were rounded to the nearest 10g. To reduce participant burden and to make the questionnaire easier to complete, we used guidelines from the British Nutrition Foundation and the British Dietetic Association to create serving size indicators in more intuitive units, such as hand sizes or tablespoons [3, 4]. Serving sizes that could be counted in units were presented as such (e.g. 3 fish fingers).

**References**

1. FSA. *Food Portion Sizes*, 3rd Ed.; TSO: Norwich, UK, 2002.
2. Fitt, E.; Mak, T.N.; Stephen, A.M.; Prynne, C.; Roberts, C.; Swan, G.; Farron-Wilson, M. Disaggregating composite food codes in the UK National Diet and Nutrition Survey food composition databank. *Eur. J. Clin. Nutr.* **2010**, *64* (Suppl. 3), S32–S36, doi:10.1038/ejcn.2010.207.
3. British Nutrition Foundation. Find Your balance—Get Portion Wise! Available online: https://www.nutrition.org.uk/healthyliving/find-your-balance/portionwise.html (accessed on 19 May 2020).
4. The British Dietetic Association. Available online: https://www.bda.uk.com/resourceDetail/printPdf/?resource=food-facts-portion-sizes (accessed on 19 May 2020).
