# Supplementary material for: Development and Reliability of the Oxford Meat Frequency Questionnaire
Source: Nutrients. 2021 Mar 12;13(3):922. doi: 10.3390/nu13030922 (PMC7999625; doi:10.3390/nu13030922)
Supplement: Supplementary file 1 [file nutrients-13-00922-s001.zip › Supplementary File S3.docx]

**Supplementary File S3:** Screenshots of the Oxford Meat Frequency Questionnaire (MFQ) hosted on Qualtrics Survey website during our reliability study


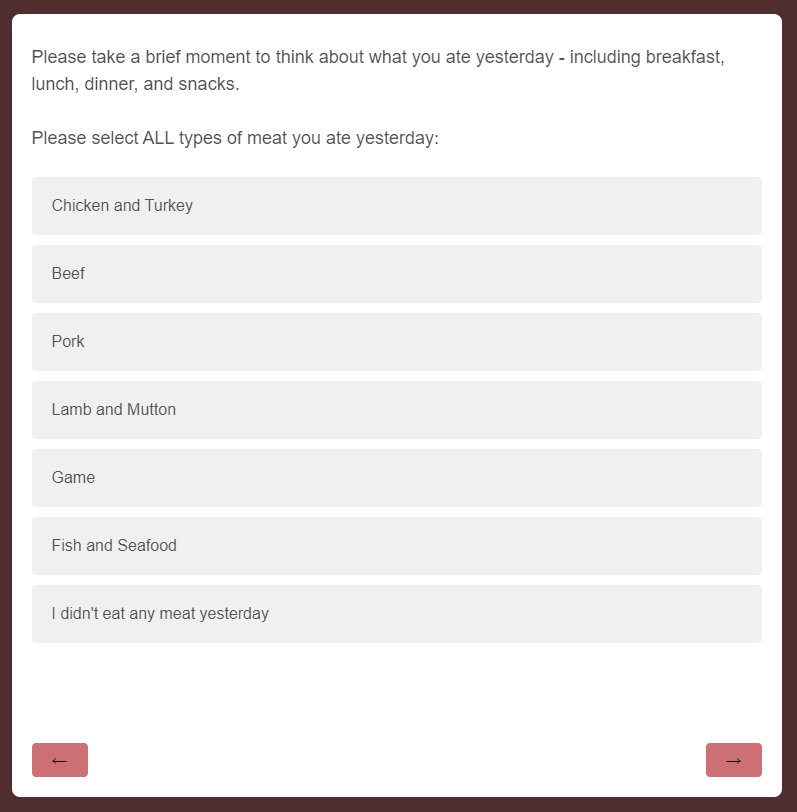


**
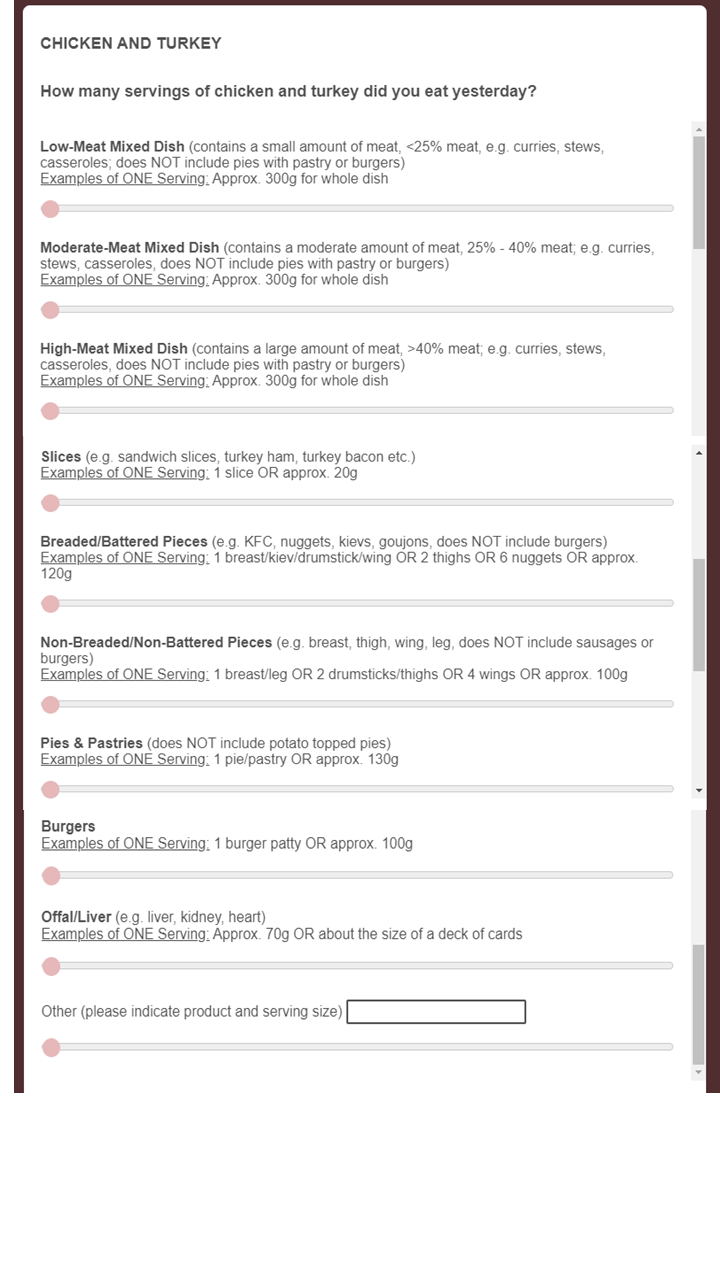
**

**
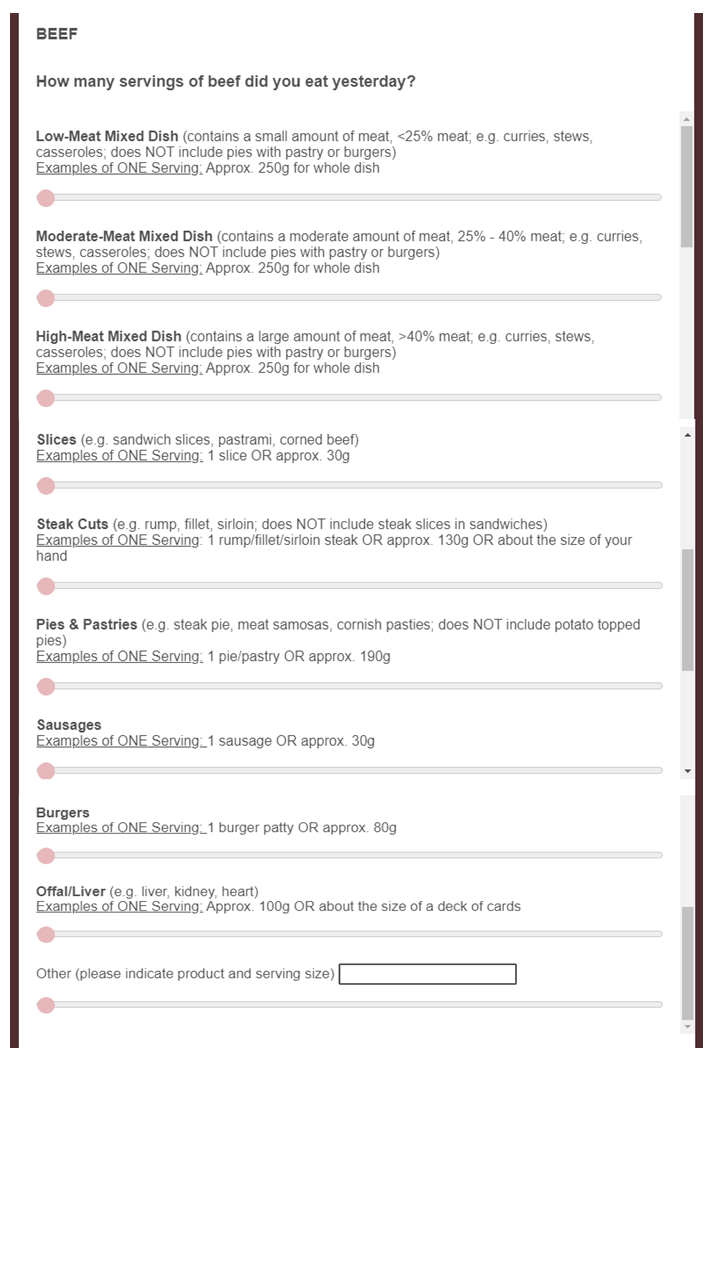
**

**
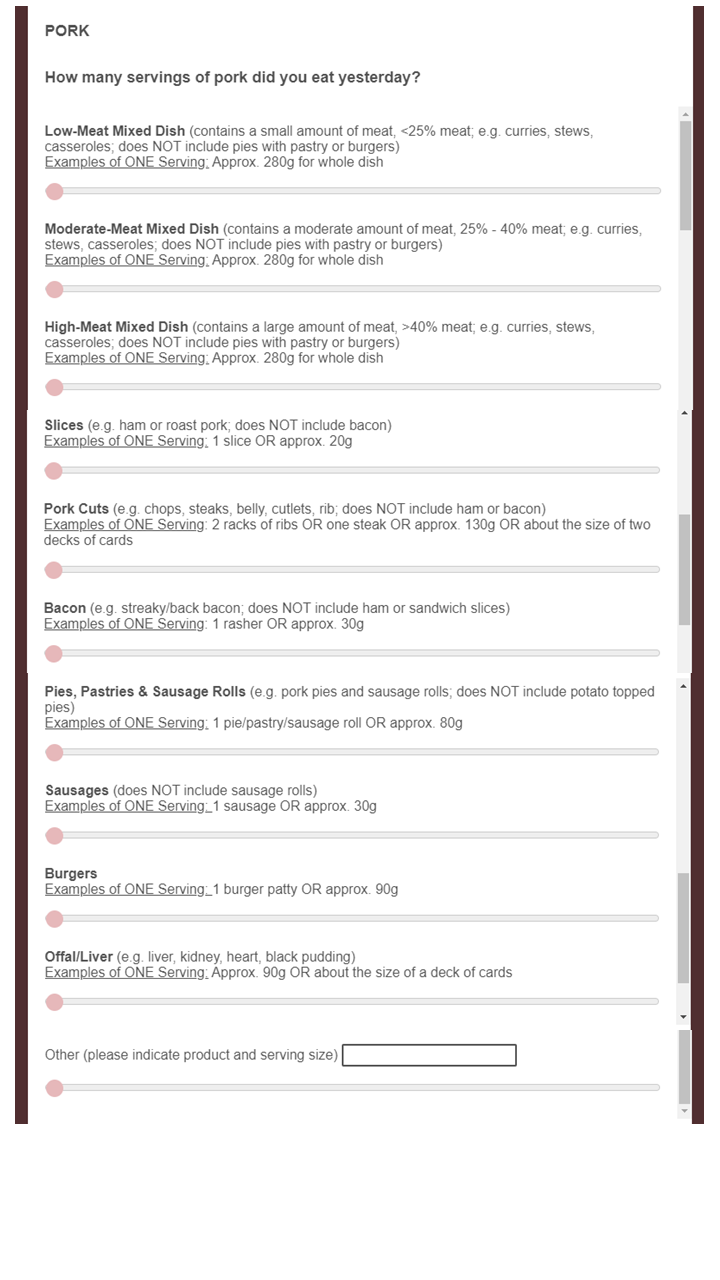
**

**
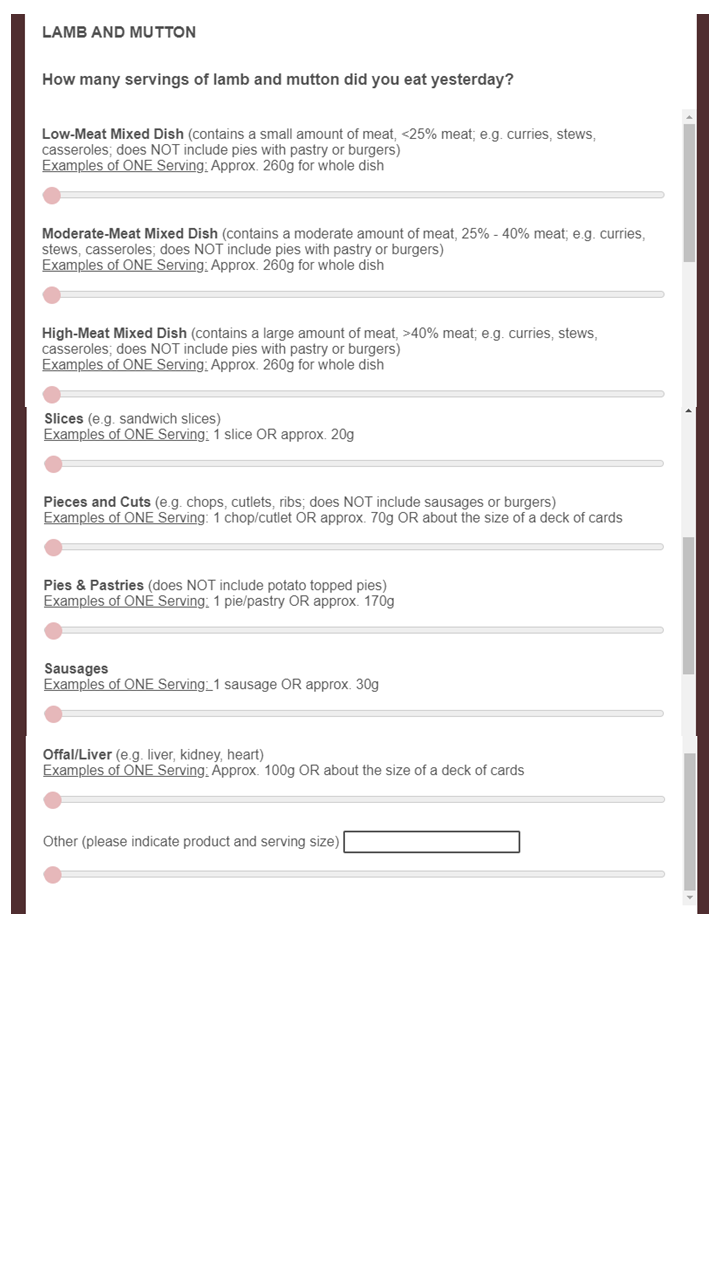
**


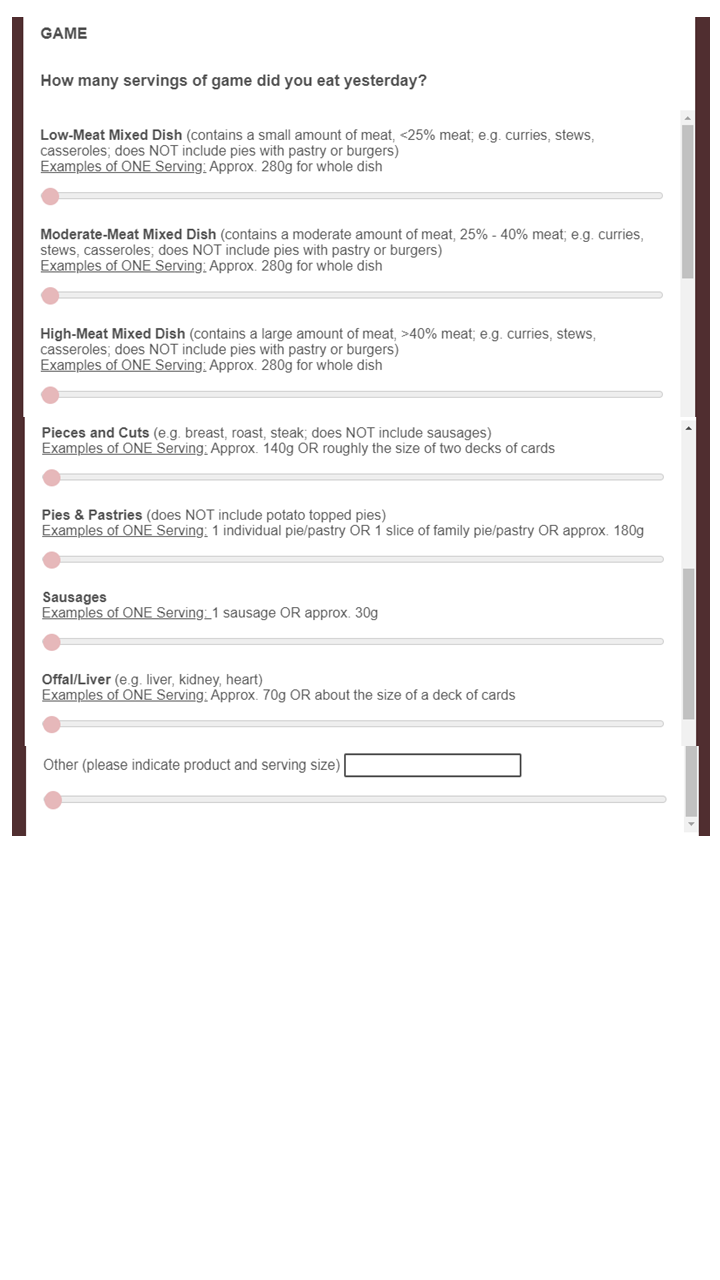


**
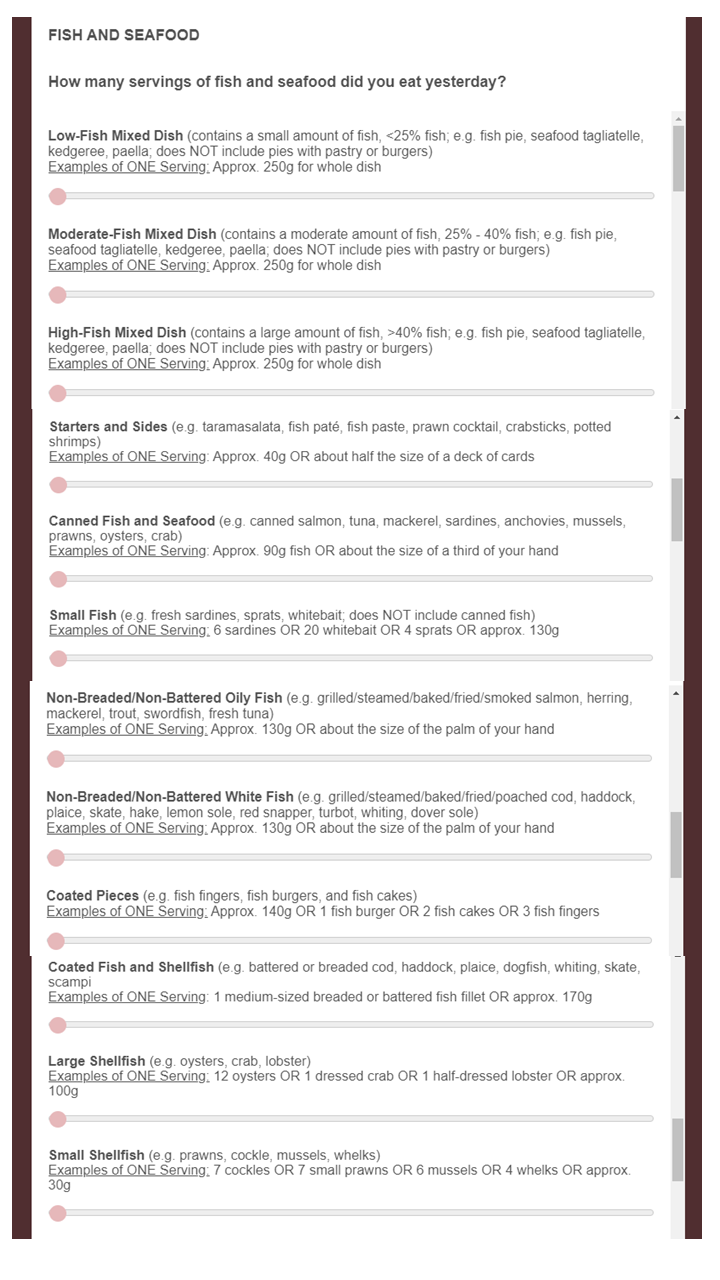
**

**
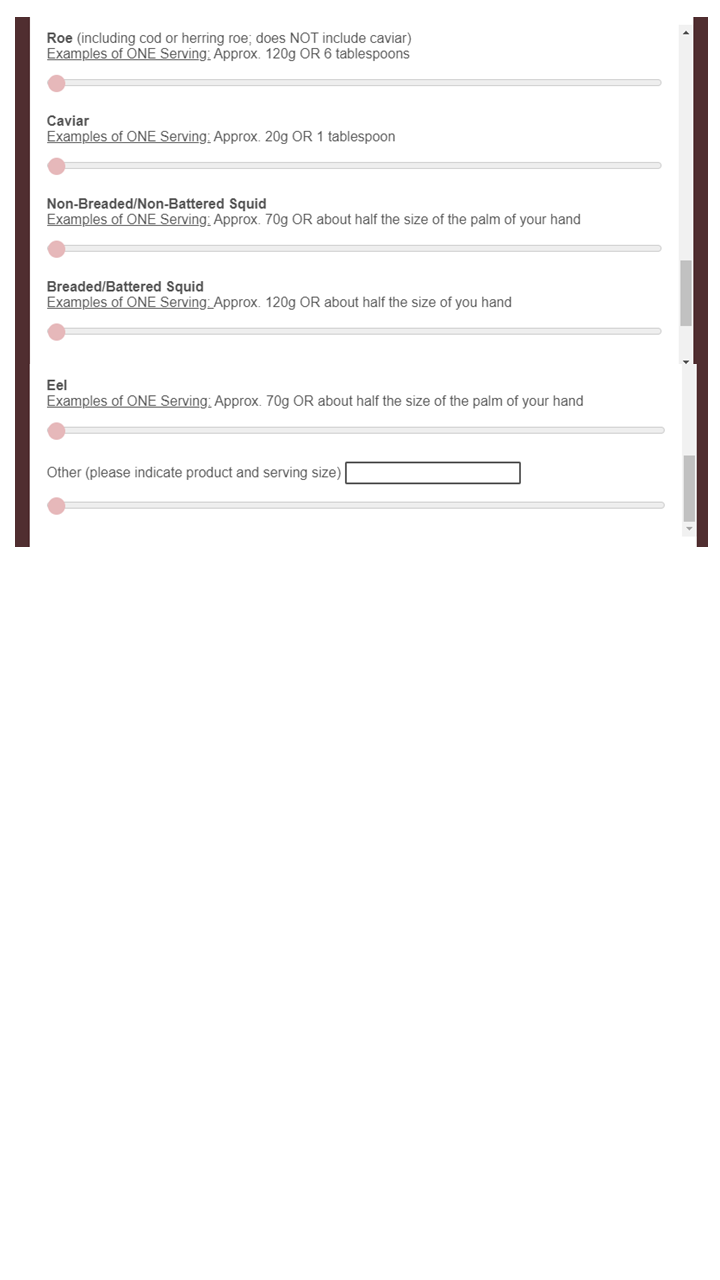
**
