# Supplementary material for: Development and Reliability of the Oxford Meat Frequency Questionnaire
Source: Nutrients. 2021 Mar 12;13(3):922. doi: 10.3390/nu13030922 (PMC7999625; doi:10.3390/nu13030922)
Supplement: Supplementary file 1 [file nutrients-13-00922-s001.zip › Supplementary File S4.docx]

**Supplementary File S4:** The Oxford Meat Frequency Questionnaires (MFQ) instructions

**Meat Intake Questionnaire Instructions**

This questionnaire is designed to record how much meat and fish you ate (including chicken, turkey, beef, pork, lamb, mutton, game, fish and seafood) on the previous day. From steak to stir fries to curry, meat and fish dishes come in various forms. We have compiled the following instructions to help you estimate how much and what kind of meat/fish you have consumed with your meals.

- You are asked to indicate the number of servings you have consumed of different meat and fish items on the previous day
- Please consider meat/fish you have eaten in and out of your home, as part of your breakfast, lunch, dinner or snack, sitting down or on the go, etc.
- Try to complete the questionnaire as accurately as possible, there are no right or wrong answers
- When completing the questionnaire, please read our description of each category carefully to know what is included
- Please use our examples of what counts as a single serving when considering how much you consumed. We provide example weights (in grams) for one serving, as well as some additional measurements (e.g. number of pieces and visual size representations such as the palm or your hand/ a deck of cards). Please use whichever serving size indicator works best for you
- At the start of the questionnaire we will ask you which of the following types of meat you ate: chicken, turkey, beef, pork, lamb, mutton, game, fish and seafood. For each meat type you choose, we will present you with a list of products. Please look through all products to see what you had
- For each meat product you've eaten, please click and drag the slider to the desired position to indicate the number of servings you had
- Please do not record the same meat item or composite meat dish in more than one category
- If you’re unsure about which category your item/dish falls into, read the descriptions carefully and estimate which category comes closest to what you have eaten
- You may find it helpful to start by jotting down on a piece of paper all the meals you had yesterday

Over the next couple of pages you can find some examples of how to complete the questionnaire and some further instructions for specific scenarios.

**How to fill in the questionnaire**

If you ate one sandwich with two slices of ham yesterday for lunch, please drag the slider to ‘2’ servings of pork slices, because one slice counts as one serving.


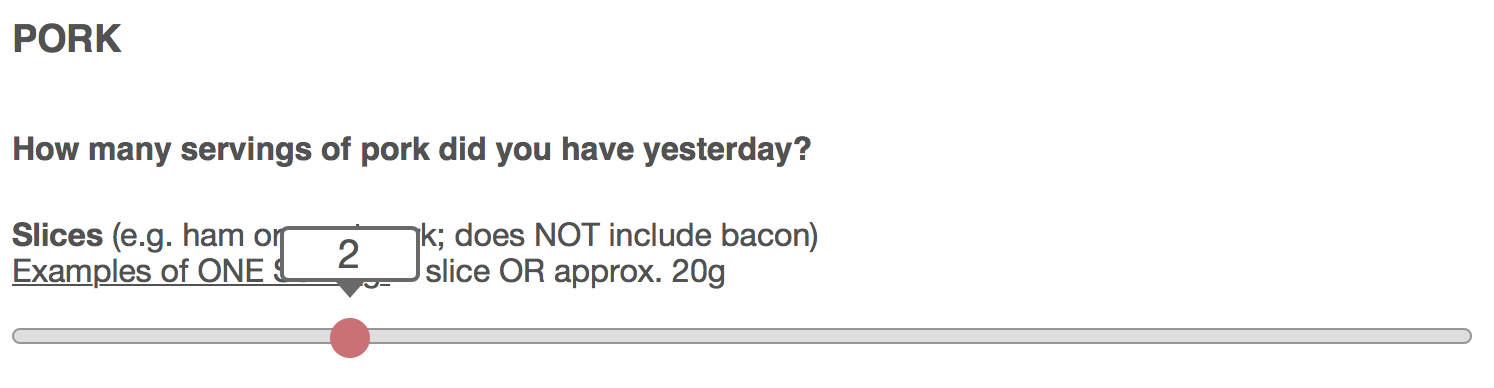


So far, so good? Let’s look at the difference between individual items and mixed dishes on the next page.

**Individual Meat/Fish Items and Mixed Dishes**

This questionnaire asks about your consumption of both individual items and mixed dishes.

**Individual Meat/Fish Items**

Individual items refer to individual pieces and cuts (e.g. breasts, legs and steak) as well as sausages, meat pies and burgers (these portion sizes can be estimated easily in counts).

**For example,** imagine you had two grilled chicken thighs with a side of roasted vegetables and rice. As the meat was separate to the side dishes and not mixed in, it wouldn’t count as a mixed dish. You would instead indicate that you had 1 serving of ‘chicken and turkey non-breaded/non-battered pieces’, because we specified that 2 chicken thighs were one serving.


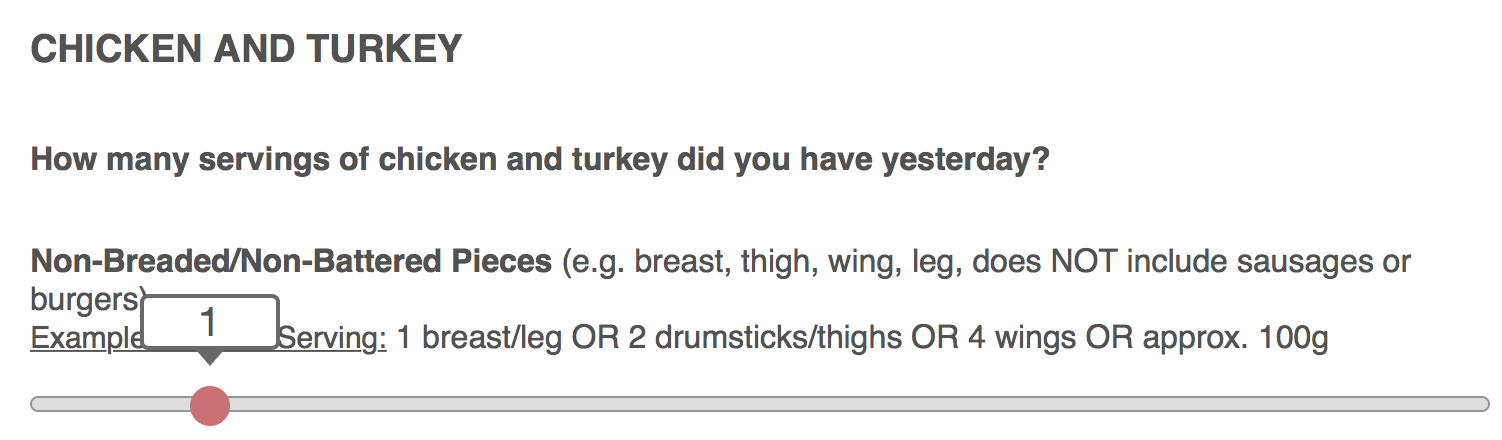


**Mixed Meat/Fish Dishes**

- Mixed dishes refer to composite dishes which include a mixture of meat and non-meat ingredients, e.g. curries, casseroles, or stews
- The portion size of a mixed dish includes the weight of ALL meat and non-meat ingredients
- We have categorised mixed dishes into three categories based on the proportion of meat they contain
- For each meat dish you consume, please estimate whether you think it contained a low, moderate or high proportion of meat

Low-Meat Mixed Dishes

- Contain a small proportion of meat (less than 25% of the dish), e.g. pepperoni pizza or a spaghetti carbonara

Moderate-Meat Mixed Dishes

- Contain a moderate proportion of meat (25-40% of the dish), e.g. chicken fajitas, beef stew containing a large amount of vegetables

High-Meat Mixed Dishes

- Contain a high proportion of meat (more than 40% of the dish), e.g. beef stew containing a small amount of vegetables

**For example,** if you had one serving of chicken curry for dinner last night, and you thought about a third was meat, you would tick ‘1’ serving of a ‘chicken and turkey moderate-meat mixed dish’.

**
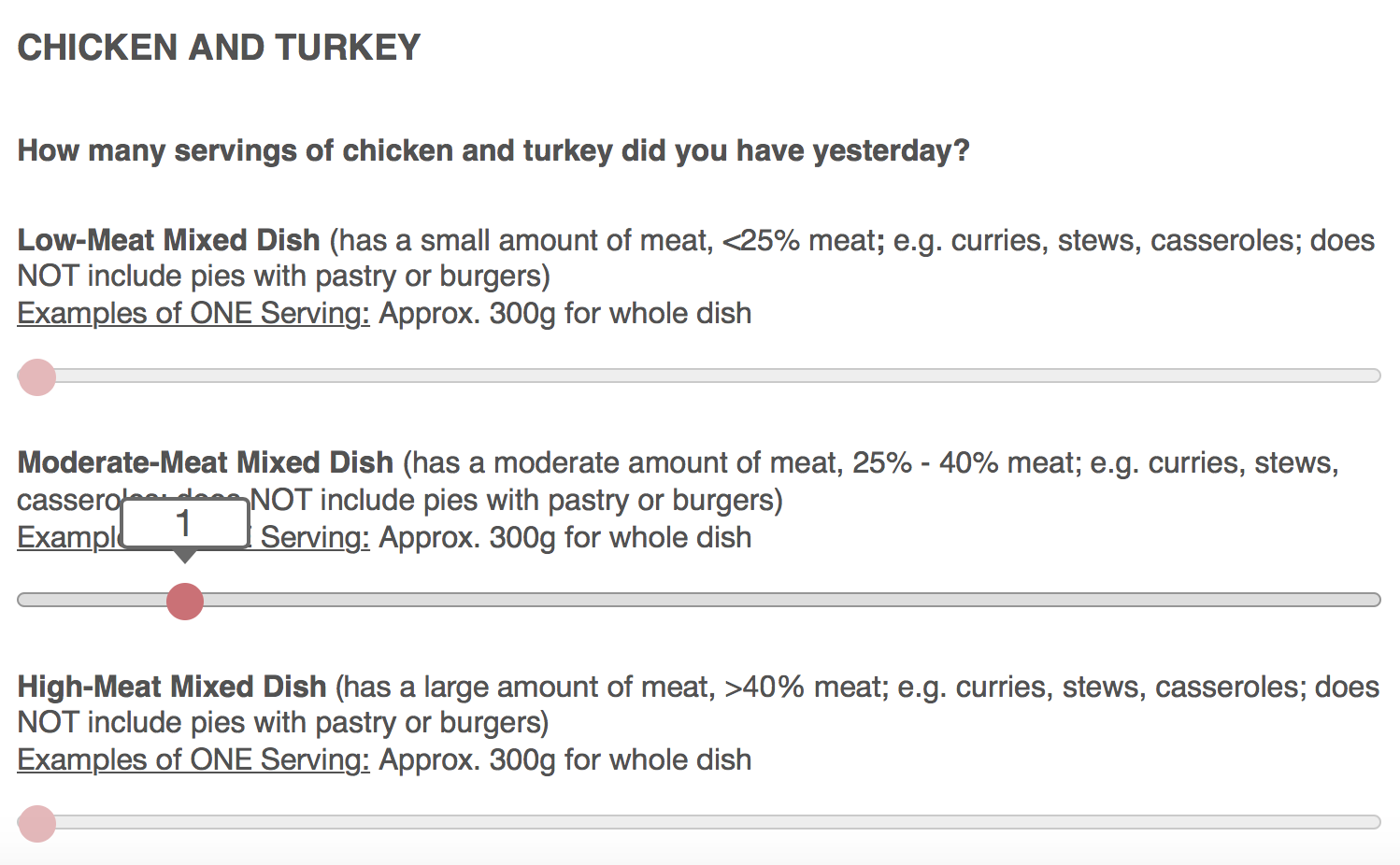
**

**Products Containing More Than One Meat Type**

- If you consume an item/dish with more than one meat type (e.g. beef and pork burger, prawn and chicken paella) please total the meat types together and record this under the animal category which has the highest contribution

**For example,** if you had two servings of chicken and prawn paella, and you estimate the total meat quantity was around 20% and it contained more chicken than prawns, you would indicate that you had 2 servings of ‘chicken and turkey low-meat mixed dish’.

**
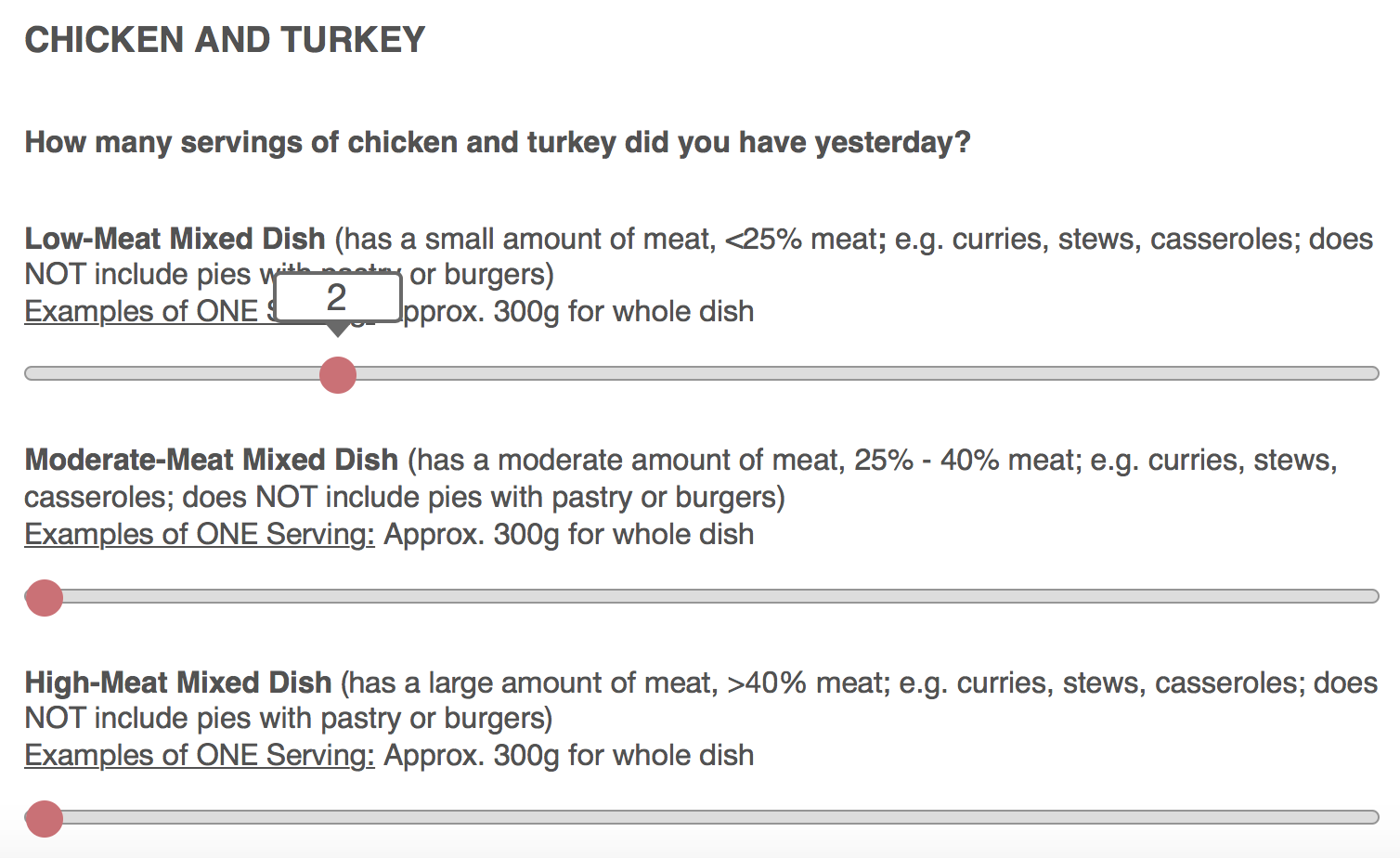
**
