# Supplementary material for: Development and Reliability of the Oxford Meat Frequency Questionnaire
Source: Nutrients. 2021 Mar 12;13(3):922. doi: 10.3390/nu13030922 (PMC7999625; doi:10.3390/nu13030922)
Supplement: Supplementary file 1 [file nutrients-13-00922-s001.zip › Supplementary File S5.docx]

**Supplementary File S5:** Bland-Altman plots for white meat, red meat, processed meat, and fish & seafood (g/day) showing the comparability of weeks 1 and 3 of the MFQ.


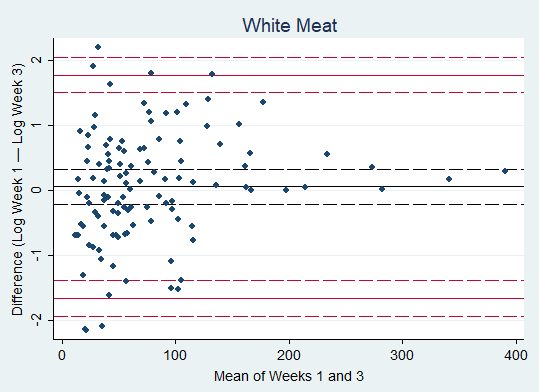

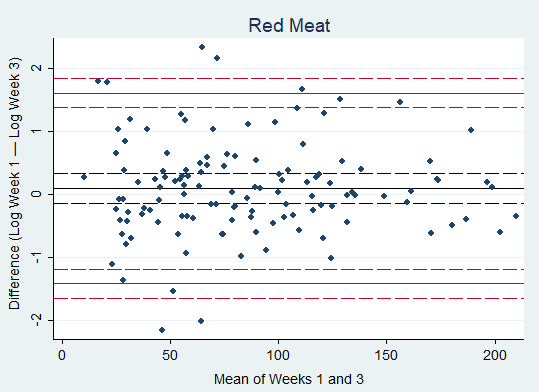


**
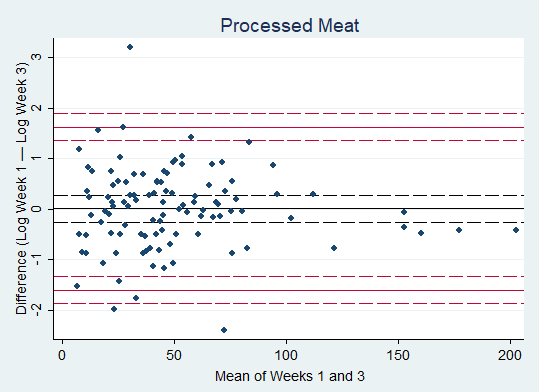

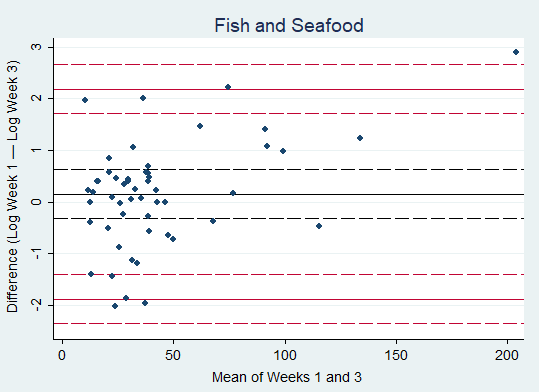
**

MFQ: Oxford Meat Frequency Questionnaire. Measurements of meat intake after log transformation of difference data plotted against original mean data. Limits of Agreement are shown as solid red lines with 95% confidence intervals (dashed lines). The mean difference (bias) is shown as the solid black line with 95% confidence intervals (dashed lines). The mean difference (bias) data is on a log scale and so is equivalent to a ratio on a linear scale.
